# Antiproliferative Fate of the Tetraploid Formed after Mitotic Slippage and Its Promotion; A Novel Target for Cancer Therapy Based on Microtubule Poisons

**DOI:** 10.3390/molecules21050663

**Published:** 2016-05-19

**Authors:** Yuji Nakayama, Toshiaki Inoue

**Affiliations:** 1 Division of Functional Genomics, Research Center for Bioscience and Technology, Tottori University, 86 Nishi-cho, Yonago, Tottori 683-8503, Japan; yujin@med.tottori-u.ac.jp; 2 Division of Human Genome Science, Department of Molecular and Cellular Biology, School of Life Sciences, Faculty of Medicine, Tottori University, 86 Nishi-cho, Yonago, Tottori 683-8503, Japan; 3 Chromosome Engineering Research Center, Tottori University, 86 Nishi-cho, Yonago, Tottori 683-8503, Japan

**Keywords:** spindle assembly checkpoint, cell death during mitotic arrest, mitotic slippage, mitotic catastrophe, tetraploid, antiproliferative fate, chemoresistance, basal autophagy, micronuclei, clustered micronuclei

## Abstract

Microtubule poisons inhibit spindle function, leading to activation of spindle assembly checkpoint (SAC) and mitotic arrest. Cell death occurring in prolonged mitosis is the first target of microtubule poisons in cancer therapies. However, even in the presence of microtubule poisons, SAC and mitotic arrest are not permanent, and the surviving cells exit the mitosis without cytokinesis (mitotic slippage), becoming tetraploid. Another target of microtubule poisons-based cancer therapy is antiproliferative fate after mitotic slippage. The ultimate goal of both the microtubule poisons-based cancer therapies involves the induction of a mechanism defined as mitotic catastrophe, which is a *bona fide* intrinsic oncosuppressive mechanism that senses mitotic failure and responds by driving a cell to an irreversible antiproliferative fate of death or senescence. This mechanism of antiproliferative fate after mitotic slippage is not as well understood. We provide an overview of mitotic catastrophe, and explain new insights underscoring a causal association between basal autophagy levels and antiproliferative fate after mitotic slippage, and propose possible improved strategies. Additionally, we discuss nuclear alterations characterizing the mitotic catastrophe (micronuclei, multinuclei) after mitotic slippage, and a possible new type of nuclear alteration (clustered micronuclei).

## 1. Introduction

**Figure 1:**
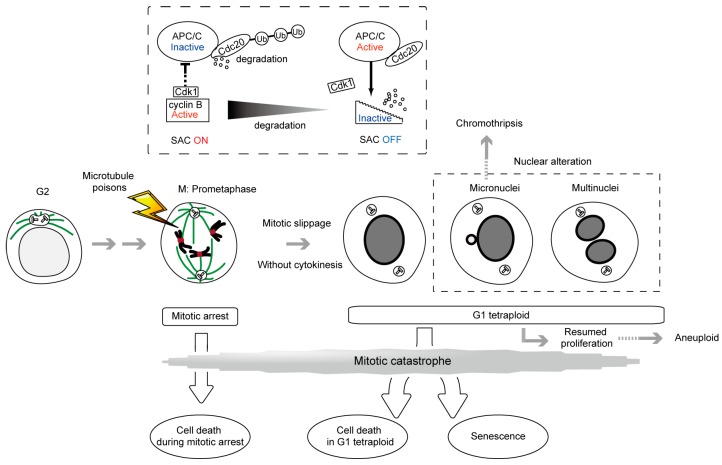
Mitotic catastrophe caused by microtubule poisons. Mitotic catastrophe is a *bona fide* intrinsic oncosuppressive mechanism that senses mitotic failure and responds by driving a cell towards an irreversible antiproliferative fate of death or senescence [3,4]. Irreversible antiproliferative processes occur during the mitotic phase in diploid cells and the tetraploid state generated after mitotic slippage. The processes governing mitotic arrest and mitotic slippage are described, with the levels of the mitotic cyclin, cyclin B1, being indicated. Nuclear alterations characterizing the mitotic catastrophe are also described. Clustered micronuclei, the third nuclear alteration, are described in Figure 4, but not in Figure 1. In the case of cancer cells whose chromosome number is frequently abnormal, diploid and tetraploid denote the cells with the original number of chromosomes before the treatment of microtubule poisons and with twice the number of chromosomes, respectively.

Microtubule poisons such as taxanes (paclitaxel, docetaxel) and Vinca alkaloids (vinblastine, vincristine, vinorelbine, vindesine, vinflunine) are used as anticancer drugs. Taxanes are microtubule stabilizing drugs that inhibit the dynamic instability of spindles and allow microtubule attachment to the kinetochores, but prevent the generation of tension across kinetochores. Alternatively, Vinca alkaloids and nocodazole (frequently used in laboratories) depolymerize microtubules and thereby prevent their attachment to the kinetochores. Both types of drugs alter microtubule dynamics and inhibit spindle function. The first aim of this review is to provide an overview of the spindle assembly checkpoint (SAC), a safeguard mechanism that induces cell cycle arrest during mitosis, and is provoked by improperly attached kinetochores to the mitotic spindle, leading to cell death inside mitosis. SAC-induced cell cycle arrest typically occurs until the attachment defect is resolved, however, microtubule poisons prolong SAC activation and mitotic arrest, which often cause cell death [1,2]. Cell death during metaphase arrest is the first target of cancer therapy by microtubule poisons (Figure 1).

One possible problem with respect to using microtubule poisons as a cancer therapy is that SAC activation and metaphase arrest are not permanent even in the presence of the drugs. Surviving cells shift towards tetraploid cells without undergoing cytokinesis (mitotic slippage), leading to secondary fates (Figure 1). The second aim of this review is to provide an overview of antiproliferative fate in tetraploid cells as a secondary target of microtubule poisons-based cancer therapies. For example, paclitaxel has recently been reported to kill cancer cells both during mitotic arrest and after mitotic slippage [5]. Both the cell death during mitotic arrest and the antiproliferative fate in the tetraploid state, form part of the recently defined ‘mitotic catastrophe’ [3,4] (Figure 1).

The mechanisms governing antiproliferative fate in tetraploid cells are not well understood compared to cell death during mitotic arrest. The third aim of this review is to expose our studies suggesting that autophagy regulation is involved in this process. 

The fourth aim is to provide an overview of another type of frequently occurring outcome resulting from treatments with microtubule poisons, gross nuclear alterations formed after mitotic slippage. One alteration leads to the emergence of micronuclei, small independent nuclei separated from the main nucleus, which arises from lagging uncondensed chromosome (Figure 1). Micronuclei can be a source of chromothripsis, a new kind of massive, yet spatially localized, genomic rearrangements process, in a single event, involved in tumorigenesis [3,4,6,7,8]. The other type of alteration yields multinuclei, a cluster of mis-segregated uncondensed chromosomes. We explain the similarities and differences between these two types of nuclear alteration, and describe a potentially new intermediate type observed in our recent study (clustered micronuclei) [9,10].

The final aim of this review is to provide novel strategies to increase the efficacy of microtubule poisons for cancer treatment.

## 2. SAC, a ‘Wait Anaphase Signal’ in the Metaphase-Anaphase Transition and Cell Death during Mitotic Arrest 

The SAC acts to maintain genome stability by delaying cell division until accurate chromosome segregation can be guaranteed [11]. Either unattached or untensioned kinetochores provoke the SAC, leading to cell cycle arrest at metaphase, preventing the mis-segregation of chromosomes by hindering the onset of anaphase through a signaling cascade that results in the suppression of the anaphase promoting complex/cyclosome (APC/C), a multi-subunit E3 ubiquitin ligase [12,13]. Once all kinetochores become stably attached to the spindle, the SAC is silenced, and cell division proceeds. Therefore, the SAC has been widely described as a ‘wait anaphase signal’ in the metaphase-anaphase transition [14]. It has been established that several evolutionally conserved proteins, including BubR1, Bub1, Bub3, Mad1, Mad2, CENP-E, and Mps1 (monopolar spindle 1) are required for SAC function [15,16].

Inhibition of spindle function by long-term treatment with microtubule poisons can have several outcomes. The first is prolonged mitotic arrest. Prolonged mitotic arrest is sometimes reversible (*i.e.*, cells can undergo normal division) upon withdrawal of microtubule poisons. 

The secondary outcome involves the execution of the cell death pathway directly from mitosis, when the levels of a mitotic cyclin, cyclin B1 that binds to cyclin-dependent kinase-1 (Cdk1), required for metaphase arrest, remain high [17] (Figure 1). This cell death pathway is characterized by the activation of caspase-2 and/or mitochondrial membrane permeabilization with the release of cell death effectors such as apoptosis-inducing factor and cytochrome c. Although the morphological aspect of apoptosis may be incomplete, this form of cell death encompasses the biochemical hallmarks of apoptosis [18,19]. It has been reported that phosphoregulation of antiapoptotic Bcl-2 proteins by Cdk1/cyclin B1 promotes cell death [20]. As an additional mechanism, phosphorylation of anti-apoptotic protein Mcl-1 by Cdk1/cyclin B1 leads to degradation of Mcl-1 and promotes apoptosis [21]. Conversely, Cdk1/cyclin B1 also phosphorylates and inactivates caspase-9, protecting cells from apoptosis during mitotic arrest. Therefore, the mechanisms by which these pro-apoptotic and pro-survival functions are regulated still need to be clarified [22,23]. The detailed signaling pathway governing the transition from mitotic arrest to apoptosis still needs to be elucidated in order to enhance cell death and improve the efficacy of microtubule poisons in cancer therapy.

The third outcome is mitotic slippage, in which cells exit prolonged mitotic arrest, escaping from cell death, while still exposed to microtubule poisons, failing cytokinesis, entering into a pseudo G1 state, and finally becoming tetraploid (Figure 1). Although the SAC activity prevents fast degradation, cyclin B1 is still slowly degraded despite chronic SAC activation. The decrease of cyclin B1 below the threshold required to maintain the mitotic state, leads to mitotic exit [24,25]. 

A weak SAC and consequent rapid slippage leads to resistance to microtubule poisons. For example, a loss of the SAC due to the suppression of Mad2 or BubR1 increases paclitaxel resistance through the blockage of Cdk1 activity [26]. It has been reported that sustained activation of Cdk1/cyclin B1 by transient overexpression of nondegradable cyclin B1 mediates pro-apoptotic signaling in response to mitotic arrest via Bcl-xL/Bcl-2 phosphorylation. This suggests that sustained activation of Cdk1/cyclin B1 may represent a promising strategy to improve the efficacy of microtubule poisons in cancer therapy [27]. 

Another strategy that has been reported recently involves blocking mitotic exit to improve the efficacy of microtubule poisons. APC/C plays an important role in the inactivation of the SAC, and the mitotic exit after prolonged SAC activation. APC/C, in association with its co-activator Cdc20, targets proteins such as cyclin B1 and securin, that inhibit separases through binding, triggering the ubiquitin-dependent destruction by the proteasome [13] (Figure 1). It has been reported that blocking mitotic exit by Cdc20 knockdown slowed cyclin B1 proteolysis and allowed more time for death initiation. Furthermore, cell death by Cdc20 knockdown did not require SAC activity [28]. As RNAi approaches indicate, the levels of Cdc20 expression must be strictly reduced to induce mitotic arrest [28,29]. Also, a small molecule inhibiting APC/C, tosyl-l-arginine methyl ester (TAME), has been reported [30]. Treatment of cells with a prodrug of TAME causes a surprisingly robust mitotic arrest and mitotic cell death. In contrast with Cdc20 knockdown, this metaphase arrest is dependent on the SAC. Considering the SAC signaling is important for the prolonged mitotic arrest, this suggests the existence of a positive feedback loop between the SAC and the APC/C that amplifies the ability of TAME-induced mitotic arrest. Another APC/C inhibitor, apcin, that blocks mitotic exit synergistically with TAME, has been developed [31]. APC/C inhibitors may be useful in combination with microtubule poisons to enhance cell death during mitotic arrest. 

Other drugs that target mitosis have also received a great deal of attention in recent years. For example, Aurora kinase A (involved in microtubule formation and/or stabilization at the spindle pole), Aurora kinase B (involved in alignment and segregation of chromosomes), Cdk1 (involved in mitotic onset and progression), CENP-E (involved in stable spindle microtubule capture at kinetochore), Eg5 (involved in centrosome separation and bipolar spindle formation), and Polo-like kinase-1 (involved in centrosome maturation and spindle assembly) are common drug targets [2]. Interestingly, Pazopanib and MLN8237 have been reported to improve antitumor activity and extended the duration to taxanes through Aurora kinase A inhibition [32,33]. Understanding the mechanisms underlying this synergy has the potential to improve the efficacy of microtubule poisons in cancer therapy.

## 3. Antiproliferative Fate after Mitotic Slippage as another Target of Cancer Therapy by Microtubule Poisons 

After mitotic slippage, tetraploid cells have three fates; they undergo cell death, become senescent, or continue dividing, although the mechanisms of fate determination remain unclear (Figure 1). 

These fates, excluding division continuation, are secondary targets of microtubule poisons-based cancer therapies, and are described as outcomes of the recently defined mitotic catastrophe, as well as cell death during mitotic arrest. According to the International Nomenclature Committee on Cell Death, the precise definition of mitotic catastrophe is a *bona fide* intrinsic oncosuppressive mechanism that senses mitotic failure and responds by driving a cell to an irreversible antiproliferative fate of death or senescence [3,4]. Therefore, the cases where mitotic arrest is followed by resumed proliferation cannot be considered as mitotic catastrophes. Such cells can generate an aneuploid, genomically unstable, and hence potentially tumorigenic progeny [17] (Figure 1). 

A single cell-based time-lapse microscopy study revealed that the propensity of cells to undergo mitotic catastrophe is not genetically predetermined. Even presumably genetically identical daughter cells frequently behave differently during the next mitosis, suggesting the involvement of other cell fate determination factors resulting in a high level of stochastic variation. Also, it was observed that duration of mitotic arrest does not dictate fate [24,34]. These insights suggest that the pathways responsible for determining these cell fates are important topics for future research aiming at developing new antiproliferative strategies for cancer cells.

An additional unknown to be solved is the process governing irreversible antiproliferative fate of death or senescence after mitotic slippage. The mechanism involved is not well understood, contrary to one governing cell death during mitotic arrest. An elucidation of these mechanisms is required to allow the improvement of the microtubule poisons’ efficacy, counteract microtubule poisons-resistance episodes in cancer cells, and suppress cancer development from normal cells. Next, based on our study on the Sirtuin (SIRT) family of NAD+-dependent protein deacetylases, we describe how the level of basal autophagy participates in inducing antiproliferative fate after mitotic slippage.

## 4. Causal Association between Basal Autophagy and Antiproliferative Fate after Mitotic Slippage

The SIRT family of NAD+-dependent protein deacetylases has been shown to play important roles in the post-translational regulation of many metabolic and cytoprotective processes [35,36]. SIRT activities depend on the availability of cofactor NAD+ and are influenced by both the cell metabolic status and the circadian rhythm [37,38]. 

Among them, the role of SIRT1 has been the most extensively studied among SIRT family members. SIRT1 regulates a wide variety of cellular processes, particularly longevity, metabolic control, genotoxic responses, and autophagy, through deacetylating transcriptional factors p53, Forkhead box protein O1 (FOXO1), FOXO3a, NF-κB, MYC, E2F1 and Hypoxia-Inducible Factor (HIF)-1α/HIF-2α, and DNA repair proteins [39]. 

SIRT2 has been reported to regulate cell cycle progression, cell differentiation, oxidative stress, blood glucose homeostasis, and programmed necrosis, via deacetylating histone H3, histone H4, FOXO1, FOXO3, RIP1, and p300 [40,41]. We identified SIRT2 as a downregulated protein in gliomas [42], and observed that exogenously expressed SIRT2 functions in the prophase to block the entry to chromosome condensation and subsequent hyperploid cell formation in glioma cell lines in response to mitotic stress [35]. To determine whether SIRT2 is involved in SAC functions, we used the colorectal cancer cell line HCT116 (a mitotic checkpoint-proficient near-diploid cell line), which is commonly employed for studies of SAC, autophagy, and SIRT2 functions [24,43,44]. As a novel function, we reported that SIRT2 downregulation, or pharmacological inhibition of its deacetylase activity, conferred resistance towards microtubule poisons to HCT116. This effect resembles that of the well-characterized SAC protein BubR1 [11]. However, whereas BubR1 downregulation abolishes SAC function, which is a requirement for subsequent mitotic catastrophe after mitotic arrest [45], SIRT2 downregulation causes mitotic arrest with longer sustained SAC activation than in the control. The significance of the delayed termination of SAC still remains unclear. Interestingly, the moment when the difference between cells with SIRT2 downregulation and the control arises, in terms of resistance towards microtubule poisons, occurs after the peak of SAC termination, suggesting that this phenomenon results from the antiproliferative fate after mitotic slippage rather than from cell death during mitotic arrest.

According to a systematic, single-cell-based approach study carried out by Gascoigne and Taylor [24], the majority of HCT116 cells also exited mitosis after prolonged arrest, but 32 % then died in the subsequent interphase, which is a fate rarely exhibited by the colorectal cancer cell line DLD-1. Our observation that SIRT2 downregulation did not confer resistance to microtubule poisons in DLD-1, further supports the hypothesis that SIRT2 downregulation confers resistance to microtubule poisons through suppression of antiproliferative fate after mitotic slippage. 

To elucidate the molecular mechanism by which SIRT2 regulates antiproliferative fate after mitotic slippage, we focused on autophagy for two reasons. The first reason is that SIRT1 was reported to positively regulate autophagy, and the second one is that autophagy has dual roles in anticancer action, as described below [46].

**Figure 2:**
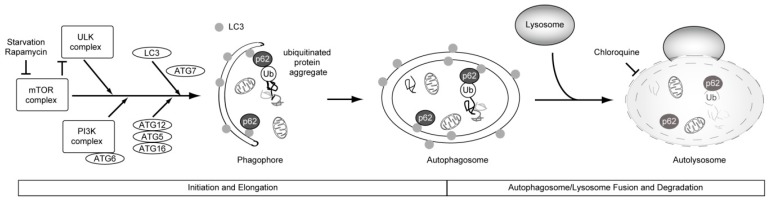
Overview of autophagy. Nutrient depletion and stress signaling, trigger autophagosome formation upon mTOR (mammalian target of rapamycin) inhibition (Initiation and Elongation). Subsequently, we describe the fusion of autophagosome with lysosome, and degradation. Autophagy is primarily non-selective. In selective autophagy, ubiquitin-binding autophagic adaptors, such as p62, play an important role in this process and selectively recognize the autophagic cargo (the inner surface of the growing phagophore), mediating its engulfment into autophagosomes, by binding to the small ubiquitin-like modifiers that belong to the LC3 (microtubule-associated protein light chain 3) family. ATG is an abbreviation for “autophagy-related”.

Autophagy is an intracellular recycling process that allows for the degradation of proteins and organelles (Figure 2). Morphologically, autophagy is characterized by the presence of autophagic vesicles (autophagosomes). Once formed, autophagosomes are delivered to the lysosome for digestion. The process of autophagy can be stimulated by nutrient starvation (induced autophagy). Nutrient-independent (often called basal) autophagy enforces intracellular quality control by eliminating toxic protein aggregates and damaged organelles. 

Although autophagy had long been considered to be a nonselective bulk degradation pathway, it was finally revealed to be at least partially selective. Substrates for selective autophagy include damaged mitochondria, intracellular pathogens, and even a subset of cytosolic proteins. Ubiquitin-binding autophagic adaptors, such as p62 and NBR1 (neighbor of BRCA1 gene 1), play an important role in this process and selectively recognize autophagic cargo, mediating its engulfment into autophagosomes by binding to the small ubiquitin-like modifiers that belong to the LC3 family [47,48,49] (Figure 2).

Autophagy plays important roles in cell survival and maintenance of cellular homeostasis during metabolic or environmental stress [50]. Dysfunction of autophagy contributes to the pathologies of many human diseases such as cancer, inflammatory diseases, and neurodegenerative diseases [51]. Accumulating evidence shows that autophagy acts as a mechanism of tumor suppression. For example, suppression of the essential autophagy genes ATG5 or ATG6 promotes tumorigenesis [52,53]. One anti-tumor function of autophagy is attributable to a surveillance mechanism for intracellular homeostasis including the removal of cytotoxic materials, such as damaged organelles and long-lived proteins. 

**Figure 3:**
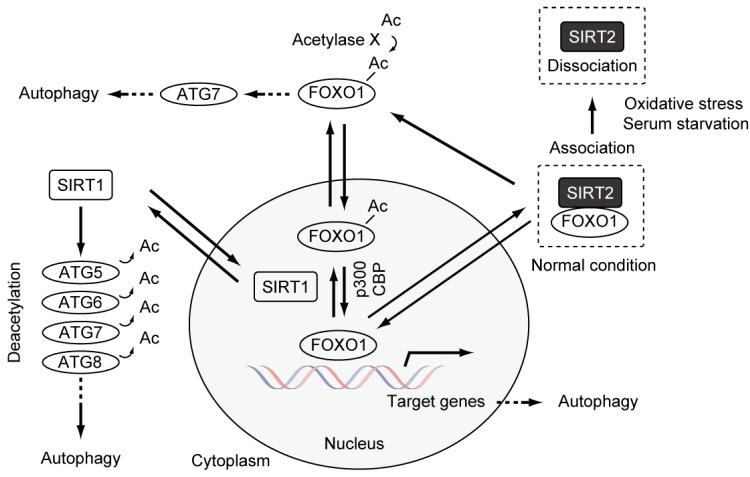
Autophagy regulation by SIRT1, SIRT2, and FOXO1.The autophagy levels are increased and decreased by SIRT1 and SIRT2, respectively. FOXO1 regulates the expression of multiple autophagy-related genes, including ATG5, ATG6, ATG8, ATG12, Rab7 (a member of RAS-related GTP-binding proteins), and Bnip3 (BCL2/Adenovirus E1B 19kDa Interacting Protein 3) [55,56,57,58,59]. There are at least six ATG8 homologues in mammalian cells, and LC3 is one of the ATG8 homologues.

SIRT1 positively regulates autophagy through its action on interacting components of the autophagy machinery, including ATG5, ATG7, and LC3. At least *in vitro*, SIRT1 can deacetylate these components [46] (Figure 3). Recently, it has been reported that deacetylation of ATG6 (Beclin1) by SIRT1 promotes autophagosome maturation [50,54]. These studies suggest a novel function for SIRT family proteins, and a possible link between the functions of SIRT1-7 and autophagy regulation.

We investigated whether SIRT2 regulates basal autophagy and whether, in that case, autophagy regulation by SIRT2 is required for antiproliferative fate after mitotic slippage. From the immunoblotting analyses of the formation of autophagosomes through the detection of two biological markers, the conversion of LC3-I to LC3-II and p62 degradation, we found that SIRT2 downregulation leads to increased basal autophagy levels independently of SAC, suggesting that SIRT2 suppresses the basal autophagy in an opposite fashion to SIRT1 [46,60]. The analysis of autophagy flux in the absence or presence of lysosomal protease inhibitors (Pepstatin A and E-64) showed that SIRT2 downregulation leads to the promotion of autophagy processes prior to lysosomal degradation, such as the autophagy induction and formation of autophagosomes [60]. Additionally, Zhao *et al*. reported that, under no stress conditions, SIRT2 suppresses autophagy via forming complexes with cytoplasmic FOXO1 transcription factor [44] (Figure 3). The interaction is disrupted upon stress-induced autophagy provoked by serum starvation or oxidative stress, leading to the increase of acetylated FOXO1 that promotes stress-induced autophagy by activating ATG7 through an unknown mechanism [44].

Subsequently, we confirmed that the increased levels of basal autophagy resulting from SIRT2 downregulation were restored back to levels that were similar to those of control cells through the downregulation of autophagy genes such as ATG5-7 and FOXO1. Importantly, the resistance to nocodazole observed in cells with SIRT2 downregulation was abolished by knockdown of these genes. These results lead to the conclusion that resistance to microtubule poisons observed in cells with SIRT2 downregulation can be ascribed to increased basal autophagy levels. Next, we sought to investigate whether this relationship between basal autophagy and resistance to microtubule poisons could be generalized beyond the SIRT2 function or SIRT2-FOXO1 axis. We used two treatments to increase the levels of basal autophagy. Treatment with rapamycin, an inhibitor of mTOR signaling [61] was used as a potential method to increase basal autophagy levels. Simultaneously, as an alternative method to increase basal autophagy levels, a mixture of EBSS (Earle's Balanced Salt Solution) and complete medium was used as a mild, but not complete starvation condition to minimize the effects of starvation on other processes [62,63].

Treatment with rapamycin or mild starvation caused increased basal autophagy without affecting cell cycle progression, conversion from G1 phase to G2/M phase in the presence of nocodazole, and the timing of SAC activation. Importantly, treatment with rapamycin or mild starvation conferred resistance to microtubule poisons through increased antiproliferative fate after mitotic slippage as observed in cells with SIRT2 downregulation. These results underscore a causal association among increased basal autophagy and resistance to microtubule poisons. 

## 5. Autophagy and Resistance to Anti-Cancer Drugs

Autophagy has been thought to be a tumor-suppression mechanism as mentioned above. On the other hand, many studies showed the causal link between increased autophagy and resistance to anticancer drugs, including paclitaxel, DNA-damaging agent cisplatin, and radiotherapy [64,65,66,67,68]. Thus, autophagy has dual roles in anticancer action. Inhibition of autophagy could be therapeutically beneficial in some cases and could be a promising target for cancer treatments. Even in the case of anticancer drugs other than microtubule poisons and radiotherapy, the precise molecular mechanism by which increased autophagy confers drug resistance remains unclear. However, it is believed that the increase in autophagic flux leads to stress restoration and metabolic homeostasis in cancer cells treated with drugs. Additionally, autophagy is assumed to provide energy for cancer cells under unfavorable conditions [69]. Also, selective autophagy can be a candidate as a mechanism to regulate antiproliferative fate after mitotic slippage. The transcriptional factors and the regulators, whose activation lead to various responses, have been listed as the targets of degradation of selective autophagy. For example, the activities of FOXO1 and GATA4 are regulated by the autophagy level [44,70]. Another well-known target is KEAP1 (Kelch-Like ECH-Associated Protein 1), a stress sensing adaptor for the Cullin3-dependent E3 ubiquitin ligase complex, that negatively regulates the NFE2L2 (Nuclear Factor, Erythroid 2-Like 2) transcription factor [71,72], whose upregulation causes paclitaxel resistance in cancer cell lines [73,74,75]. 

Interestingly, it is known that many anticancer drugs stimulate autophagy by inhibiting the phosphatidylinositol 3-kinase (PI3K)/mTOR and AMP-activated protein kinase (AMPK) signaling pathways [76] (Figure 3). Recent studies reported that the treatment with paclitaxel induced upregulation of ATG6 and TXNDC17 (Disulfide reductase) expression, and an increase in autophagosome formation. The downregulation of ATG6 or TXNDC17 attenuated the activation of autophagy and paclitaxel resistance. Although fate after mitotic slippage was not examined, these studies also supported a causal link between autophagy and paclitaxel resistance in cancer [77,78]. Additionally, the concept by which the treatment of microtubule poisons can induce the increase of basal autophagy via upregulation of autophagy genes, leading to resistance to microtubule poisons, is novel.

As a strategy to improve microtubule poisons-based cancer therapies, accumulating evidence suggests that chloroquine and its derivative hydroxychloroquine, which are autophagy inhibitors, sensitize cancer cells to radiation and anticancer drugs [69,79]. These inhibitors decrease lysosomal function, thus hindering the supply of energy through the disruption of the autophagy process. Furthermore, it has been reported that pharmacological screening of selective killers of tetraploid HCT116 clone cells led to the identification of resveratrol and aspirin as potent antitetraploids [80]. Both resveratrol and aspirin function as activators of AMPK, leading to decreased autophagy [80]. These insights support the idea that the suppression of autophagy is a promising strategy to promote antiproliferative fate in tetraploid cells. 

## 6. Potential Candidate Strategies to Improve the Efficacy of Microtubule Poisons in Cancer Therapy, Focusing on Antiproliferative Fate after Mitotic Slippage

We explain two biological phenomena that may give clues to delineate the mechanism by which autophagy regulates antiproliferative fate after mitotic slippage. These two phenomena are ‘G1 tetraploidy checkpoint’ and ‘mitosis skip followed by tetraploid senescence’, both of which occur in normal mammalian cells. Although the mechanical kinship between these two phenomena and antiproliferative fate after mitotic slippage in cancer cells is not clear, arising tetraploid cells might be conserved in both normal and cancer cells. Studying these two phenomena in normal cells will provide clues towards raising a hypothesis about antiproliferative fate after mitotic slippage in cancer cells.

In response to mitotic slippage, cell fusion, endoreduplication, or cytokinesis failure, normal cells stably arrest in G1 phase with 4C DNA content [81]. Cells that lack functional p53 have an increased ability to re-enter the cell cycle with another round of DNA replication [82]. This p53-dependent arrest of tetraploid cells is referred to as the G1 tetraploidy checkpoint [83]. Although this concept is appealing, it remains controversial. For example, some reports indicate that p53 activation might actually result from abnormal microtubules rather than from tetraploidy itself [81,84]. Furthermore, some normal tetraploid or polyploid cells are capable of proliferation; for instance, normal hepatocytes of the liver [85,86].

Although the involvement of p53 in G1 tetraploidy checkpoint remains controversial, this concept prompted us to examine whether the increased autophagy by SIRT2 downregulation suppresses antiproliferative fate after mitotic slippage through p53 function in HCT116 cells. We observed that SIRT2 downregulation conferred resistance to microtubule poisons in p53-/- HCT116 cells and that simultaneous downregulation of ATG5 restored the sensitivity. This suggests that autophagy may regulate, at least partially, antiproliferative fate in tetraploid cells in a p53-independent manner at least in HCT116 cells. Since p53 is frequently inactivated in tumors, this provides good perspectives regarding further research on mediators under the control of basal autophagy, whose regulation may efficiently induce antiproliferative fate after mitotic slippage.

The other event is mitosis skip followed by tetraploid senescence that occurs in human normal diploid cells exposed to various senescence-inducing stimuli such as activated oncogenes, reactive oxygen species (ROS), DNA damage, and replicative senescence. Mitosis skip is mediated by p53-dependent premature activation of APC/C-Cdh1 that controls mitotic exit in late mitosis and G1 maintenance, and pRb family-dependent transcriptional suppression of mitotic regulators such as cyclin B1 and Cdc20. Importantly, p16, an inhibitor of CDK4 and CDK6 is required for G1 tetraploid arrest, but not for mitosis skip [87]. Thus, p16 may be a promising target for the induction of antiproliferative fate in microtubule poisons-treated cancer cells (see also Figure 5). 

## 7. Micronucleus Formation as a Morphological Feature of Mitotic Catastrophe and the Link to Genomic Instability

Mitotic catastrophe is characterized by unique nuclear alterations including the formation of micro- and/or multinuclei [3,4,6]. Micronuclei can also be formed after mitotic slippage provoked by treatment by microtubule poisons and are detected as one or a few smaller nuclei, independent from the main nucleus. A micronucleus can arise from a whole or broken chromosome, which gets segregated improperly during mitosis (lagging chromosome) [88,89,90]. Micronuclei can also arise through a variety of genotoxic stresses including irradiation and DNA damaging agents [91,92]. Thus, micronuclei have been widely considered as indicators of chromosome instabilities. While the presence of micronuclei is thought to be related to apoptosis [92], they have also been associated with cancer propagation. If the latter is true, micronuclei may be used as a biomarker for cancer and micronuclei assay could be used for risk prediction [93]. Multinuclei are formed from clusters of mis-segregated uncondensed chromosome resulting from cytokinesis failure. Although multipolar mitosis contributes to the formation of multinuclei but not micronuclei [92], the difference in the formation of micronuclei and multinuclei is not clear.

**Figure 4:**
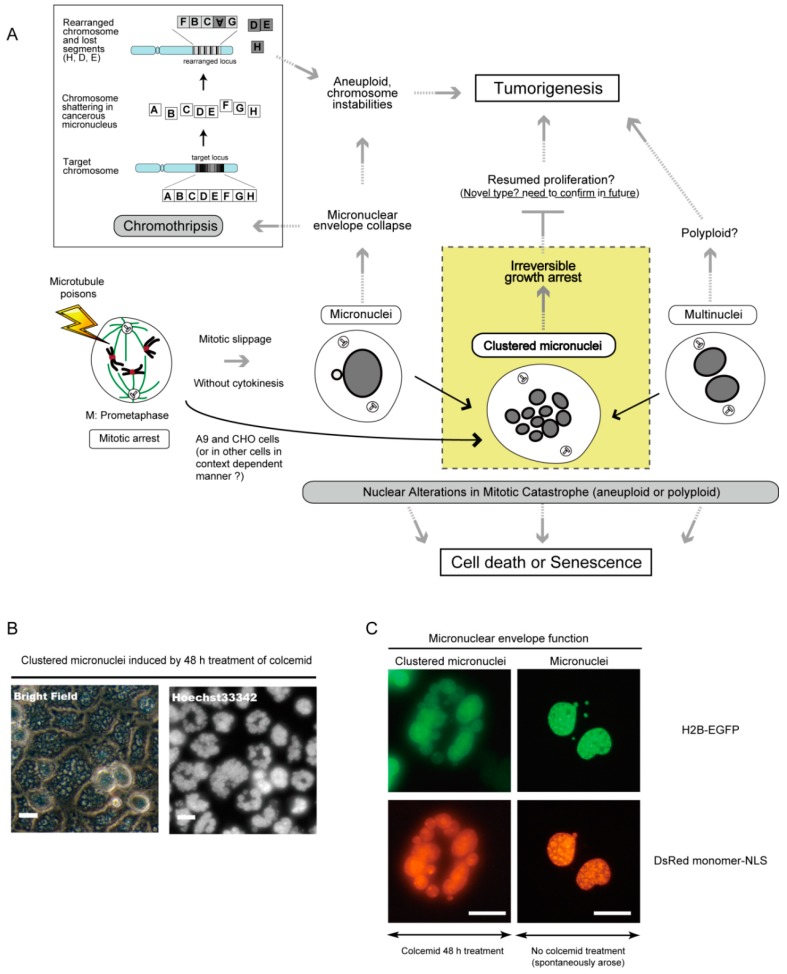
Two conventional nuclear alterations, and a new type, in mitotic catastrophe. (**A**) Conventional micronuclei can be a source for chromothripsis, which may lead to chromosome instabilities and tumorigenesis. In contrast, clustered micronuclei are thought to have a low chance to cause such genomic instabilities, since they could reach irreversible growth arrest and possess functional micronuclear envelopes as shown in Figure 4C. Cells with multinuclei may also contribute to tumorigenesis. Black arrows indicate a challenging, but not established, beneficial effect converting any of the nuclear alterations into clustered micronuclei. For details, see the text; (**B**) Morphological features of clustered micronuclei in mouse A9 cells are shown. DsRed monomer-NLS allows the production of monomeric DsRed protein fused to a nuclear localization signal. After 48 h of treatment with colcemid, clustered micronuclei are visible via light microscopic observation. Scale bars; 20 μm; (**C**) Each micronucleus within clustered micronuclei possesses a functional micronuclear envelope, but not micronuclei. Note that the micronuclei showed in the right panels arose spontaneously and are observed in a giant cell that has two large nuclei with three independent very small nuclei without functional nuclear envelope. Scale bars; 20 μm.

Recent studies have suggested that the collapse of the micronuclear envelope in micronuclei can be a source of chromothripsis (a new kind of massive genomic rearrangement), a novel mechanism by which the micronuclei formation promotes tumorigenesis [94,95] (Figure 4A). Chromothripsis is usually restricted to a single chromosome or a few chromosomes, and is thought to occur only once during tumorigenesis [94,96]. Chromothripsis is detected in many cancers by whole-genomic sequence analysis, and currently available data suggest a strong correlation between chromothripsis and poor prognosis [97,98].

**Figure 5:**
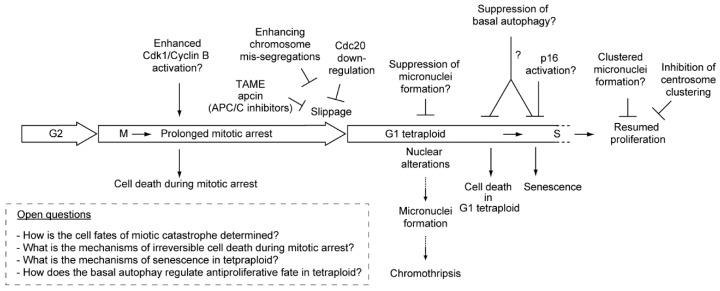
Potential strategies to improve the efficacy of microtubule poisons in cancer therapy (for details, see the text).

This description seems to be inconsistent with the definition of mitotic catastrophe as an oncosuppressive mechanism. The previous single cell-based time-lapse microscopy study suggested that the outcomes of microtubule poisons’ treatment can vary even among daughter cells, and that the formation of micronuclei may confer the potential to elicit the cell death pathway after the induction of mitotic catastrophe [25]. Another study reported that apoptotic and necrotic traits had also been observed in cells with multinuclei [5,92]. These reports support the idea that the micronucleation itself can be an oncosuppressive phenomenon, depending on the context. Reducing the chance of chromothripsis by better understanding it would help to improve the efficacy of microtubule poisons in cancer therapy (see also Figure 5).

## 8. Clustered Micronuclei Formation as a Possible New Type of Nuclear Alteration in Mitotic Catastrophe

Both micronuclei and multinuclei are nuclear alterations characterizing mitotic catastrophe. Recently, we reported a possible new type of micronuclei [10], and refer to it as ‘clustered micronuclei’ in this review. We provide an overview of clustered micronuclei with its oncosuppressive features.

Clustered micronuclei are composed of small and numerous micronuclei in a large population of mouse A9 and hamster CHO cells following treatment with colcemid, a microtubule poison (Figure 4B). No main nucleus is apparent. This is unlike micronuclei formation. The process of clustered micronuclei formation reoccurs during two or three rounds of DNA replication that were followed by mitotic slippage. The number of micronuclei increases, and the size of each micronucleus decreases, during the recurrent process.

Disassembly and reassembly of the micronuclear envelope (nuclear envelope cycle) of each micronucleus synchronously occur during every round of clustered micronuclei formation at the onset of mitosis and at the time when cells exit mitosis, shifting to a pseudo G1 phase, respectively. DNA replication in each micronucleus is also synchronously observed, unlike in micronuclei and the main nuclei [10]. Thus, it is thought that each micronucleus within clustered micronuclei possesses a functional micronuclear envelope. 

To assess the functionality of the nuclear envelope of the clustered micronuclei, we introduced a DsRed (Discosoma sp. red fluorescent protein) monomer fused to a nuclear localizing signal (DsRed monomer-NLS) and EGFP (enhanced green fluorescent protein) fused to Histone 2B (H2B-EGFP) into mouse A9 cells, and treated these cells with colcemid to form clustered micronuclei. We observed that the DsRed monomer-NLS signals completely merged with the H2B-EGFP signals within clustered micronuclei (Figure 4C). Conversely, the same experiment carried out on A9 cells with micronuclei showed no, or little, nuclear localization of DsRed monomer-NLS, as previously reported [99]. This suggests that proper nuclear envelope function was maintained in clustered micronuclei unlike in micronuclei. Thus, clustered micronuclei may be safer than micronuclei, since they seem to have less chance to be a source of chromothripsis and micronuclear envelope collapse, leading to genomic instability, than micronuclei.

Also we noticed that A9 cells with clustered micronuclei had lost their growth ability, even after the removal of colcemid [10]. After two rounds of recurrent formation of clustered micronuclei, a large proportion of A9 cells became flattened and ceased DNA replication. These A9 cells did not undergo cell death and remained attached to the culture dish, displaying a flattened morphology in the normal medium. It remains unclear whether this state is a senescence-like growth arrest, which is also observed in cancer cell lines [100,101,102].

These data suggest that the formation of clustered micronuclei is a new type of mitotic catastrophe leading to irreversible growth arrest and has oncosuppressive features. Although the molecular mechanisms underlining irreversible growth arrest in A9 cells with clustered micronuclei is unknown, it is plausible that the gross nuclear alteration itself contributes to the growth arrest, as in the reported cases of micronuclei and multinuclei [4,103,104]. Further research needs to be done to examine whether cells having clustered micronuclei fail to yield tumor formation, particularly in mouse transplantation assays, and cause genomic instability.

Another open question is relative to the mechanism of clustered micronuclei formation and the common points and differences among the three types of nuclear alteration. Since clustered micronuclei are thought to have less chance to cause genomic instability than micronuclei, the inductive conversion from micronuclei to clustered micronuclei may be a strategy for effective cancer cell killing by microtubule poisons (see also Figure 5).

## 9. Conclusions and Future Prospects

As mentioned above, several possible strategies could improve the efficacy of microtubule poisons, including inducing apoptosis from SAC signaling, prolonging mitotic arrest, blocking mitotic exit, enhancing the antiproliferative fate of tetraploid cells, and inducing the formation of clustered micronuclei instead of micronuclei formation (Figure 5).

This section introduces other two strategies focusing on tetraploid cells, which are not mentioned in the other sections, but seem attractive for microtubule poisons-based cancer therapy. One is relative to the inhibition of centrosome clustering. Centrosome clustering allows bipolar division in tetraploid cells containing an extra number of centrosomes. Since tetraploid cells have two centrosomes that can be replicated during continuing division, such cells can underdo multipolar mitosis at the next mitosis. This generates three or more daughter cells, and thus can be lethal for most of them. However, bipolar mitosis allowing accurate segregation of chromosomes also occurs in cancer cells by centrosomal clustering, leading to the functional inactivation of supernumerary centrosomes. Potent inhibitors of centrosomal clustering in malignant cells have been developed [105,106], and the combination of these drugs with microtubule poisons may be a promising strategy. Recently, it has been reported that the CEP215-HSET complex promotes the clustering of extra centrosomes into pseudo-bipolar spindles, thereby ensuring viable cell division in cancer cells [107]. CEP215 and HSET are the microcephaly- and primordial dwarfism-linked centrosomal protein and the minus end-directed microtubule motor protein, respectively.

The other strategy relates to the enhancement of chromosome mis-segregations. It has been reported that partial reduction of essential mitotic checkpoint proteins made tetraploid cells remarkably more sensitive to low doses of paclitaxel [108]. This effect was thought to be associated with enhanced chromosome mis-segregations but not the SAC weakening, since it was obtained through reduction of Mps1 or BubR1, having dual roles in both checkpoint activation and chromosome alignment, but not through reduction of Mad2, affecting solely the mitotic checkpoint [108]. Untransformed human fibroblasts with reduced Mps1 levels were not sensitized to sublethal doses of paclitaxel, showing that enhancing chromosome mis-segregations can selectively kill tumor cells in the presence of paclitaxel. Thus, enhancing chromosome mis-segregations chromosome alignment can be a promising strategy to improve the efficacy of microtubule poisons.

Increasing the efficacy of microtubule poisons requires further elucidation of the mechanisms governing the mitotic catastrophe and the dynamics of both chromosomes and centrosomes. How are the SAC activation and mitotic arrest periods determined? How is the apoptosis signal induced by mitotic arrest? How can the senescence be induced in cancer cells? How are the centrosome clustering and de-clustering regulated? Future studies focusing on these issues will help, not only in the use of microtubule poisons in cancer therapies, but also in preventing normal cells from becoming tumorigenic.

## Abbreviations

The following abbreviations are used in this manuscript:

APC/C	Anaphase promoting complex/cyclosome
SAC	Spindle assembly checkpoint
TAME	Tosyl- l-arginine methyl ester
